# Do TETRA (Airwave) Base Station Signals Have a Short-Term Impact on Health and Well-Being? A Randomized Double-Blind Provocation Study

**DOI:** 10.1289/ehp.0901416

**Published:** 2010-01-14

**Authors:** Denise Wallace, Stacy Eltiti, Anna Ridgewell, Kelly Garner, Riccardo Russo, Francisco Sepulveda, Stuart Walker, Terence Quinlan, Sandra Dudley, Sithu Maung, Roger Deeble, Elaine Fox

**Affiliations:** 1 Department of Psychology and Centre for Brain Science; 2 Department of Computer Science and Electronic Engineering and; 3 Department of Electronic Systems Engineering, University of Essex, Colchester, United Kingdom; 4 Department of Electrical, Computer and Communications Engineering, South Bank University, London, United Kingdom; 5 Lensfield Medical Practice, Cambridge, United Kingdom

**Keywords:** electromagnetic fields, electromagnetic hypersensitivity, electrosensitivity, idiopathic environmental intolerance, mobile phone

## Abstract

**Background:**

“Airwave” is the new communication system currently being rolled out across the United Kingdom for the police and emergency services, based on the Terrestrial Trunked Radio Telecommunications System (TETRA). Some police officers have complained about skin rashes, nausea, headaches, and depression as a consequence of using their Airwave handsets. In addition, a small subgroup in the population self-report being sensitive to electromagnetic fields (EMFs) in general.

**Objectives:**

We conducted a randomized double-blind provocation study to establish whether short-term exposure to a TETRA base station signal has an impact on the health and well-being of individuals with self-reported “electrosensitivity” and of participants who served as controls.

**Methods:**

Fifty-one individuals with self-reported electrosensitivity and 132 age- and sex-matched controls participated in an open provocation test; 48 sensitive and 132 control participants went on to complete double-blind tests in a fully screened semianechoic chamber. Heart rate, skin conductance, and blood pressure readings provided objective indices of short-term physiological response. Visual analog scales and symptom scales provided subjective indices of well-being.

**Results:**

We found no differences on any measure between TETRA and sham (no signal) under double-blind conditions for either controls or electrosensitive participants, and neither group could detect the presence of a TETRA signal at rates greater than chance (50%). When conditions were not double blind, however, the self-reported electrosensitive individuals did report feeling worse and experienced more severe symptoms during TETRA compared with sham.

**Conclusions:**

Our findings suggest that the adverse symptoms experienced by electrosensitive individuals are due to the belief of harm from TETRA base stations rather than to the low-level EMF exposure itself.

“Airwave” is the new communication system currently being rolled out across the United Kingdom for the police and emergency services. It is based on Terrestrial Trunked Radio Telecommunications System (TETRA), which offers enhanced digital communication that improves emergency services’ performance and public safety. However, a growing number of concerns have been raised about the possible adverse health effects related to this new technology. For example, some police officers have complained about skin rashes, nausea, sleeplessness, headaches, and depression as a consequence of using their Airwave handsets (Farrel 2002; Police Federation News 2005). Others have complained about unusual symptoms, which they attribute specifically to TETRA base station signals. In addition, there is a small subgroup of persons in the general population who believe they suffer from “electrosensitivity.” As the term suggests, sufferers believe that their symptoms are caused by devices emitting electromagnetic fields (EMFs), and anecdotal evidence points to TETRA as the source of some of these symptoms. Prevalence rates for self-reported electrosensitivity vary. For example, in Iran the prevalence rate is “considerably low” (Mortazavi et al. 2007), whereas in Germany it is 10.3% (Blettner et al. 2009). Sufferers complain of reduced quality of life (Irvine 2005) and report poorer health and well-being compared with people without “idiopathic environmental intolerance with attribution to electromagnetic fields” (IEI-EMF) (Eltiti et al. 2007a). Some evidence indicates a possible general autonomic nervous system dysfunction in electrosensitive individuals (Lyskov et al. 2001a, 2001b; Sandström et al. 2003).

To address the question of whether TETRA communication technology has any impact on health and well-being, the U.K. Home Office has funded a large-scale epidemiological study to assess whether Airwave handsets have an impact on the health of police officers, and the results of this study are expected in 2018 (Airwave Health Monitoring Study 2009). In addition, the present randomized double-blind study was funded to assess the short-term impact of TETRA signals on indices of health and well-being in both self-reported electrosensitive individuals and members of the general public who do not report problems with mobile phone technologies. Little scientific information is currently available on the possible impact of TETRA on health. Randomized double-blind studies on Global System for Mobile Communication (GSM) and Universal Mobile Telecommunications System (UMTS) have reported no reproducible evidence of adverse health effects for either healthy controls or individuals with perceived electrosensitivity (Eltiti et al. 2007a; Regel et al. 2006; Rubin et al. 2006). Moreover, neither electrosensitive nor healthy individuals can detect the presence of EMFs at rates greater than chance (Eltiti et al. 2007a; Kwon et al. 2008; Regel et al. 2006; Rubin et al. 2006, 2010; Zwamborn et al. 2003). In a recent study using functional magnetic resonance imaging Landgrebe et al. (2008) showed that the subjective perception of symptoms was triggered by the anticipation of being exposed to an EMF. This finding led to the implication that these symptoms are due to beliefs and anticipation of harm rather than to the EMFs themselves. Consequently, the World Health Organization has suggested that terms such as “electrosensitivity” should be replaced by IEI-EMF to acknowledge the absence of evidence for a causal connection between EMFs and reported symptoms (Hansson Mild et al. 2006).

The health concerns regarding TETRA handsets and base stations are predominantly focused on the 17.6-Hz pulse frequency feature and its possible impact on biological systems [Advisory Group on Non-Ionising Radiation (AGNIR) 2001]. In 2001, the AGNIR reviewed all of the available evidence, including calcium efflux, brain waves, and epilepsy. The group concluded that any effect on biological systems would require a nonlinear biological interaction that operated on the time scale of the carrier frequency that could demodulate the amplitude-modulated component (Challis 2005; NRPB 2001; Valberg et al. 2007). Given this, the AGNIR concluded that TETRA was highly unlikely to pose a health risk but nevertheless recommended that well-designed laboratory studies be conducted (NRPB 2001). Only two studies investigating TETRA technology have been published since the AGNIR report was released. Green et al. (2005) found no evidence that TETRA handsets produced significant changes in calcium physiology in the brain as a consequence of TETRA exposure, whereas Barker et al. (2007) reported that neither GSM nor TETRA handset signals affected blood pressure or any other physiological parameter. The present study is the first report of the short-term health effects of TETRA base stations.

The aims of the present study were to determine whether symptoms reported by IEI-EMF sufferers are caused by short-term exposure to radiofrequency (RF) EMFs as produced by TETRA base station signals and to determine whether exposure to these signals affects a selection of the adult population that do not report sensitivity to EMFs. We tested TETRA base station signals on both healthy controls and IEI-EMF sufferers under open provocation and double-blind conditions while measuring a range of objective and subjective indicators of well-being, as well as the participants’ ability to detect the presence or absence of the signal.

## Materials and Methods

### Participants

All participants completed the Electromagnetic Hypersensitivity Questionnaire (EHQ) (Eltiti et al. 2007b) prior to testing, which provided an assessment of their current state of health and whether participants attributed their symptoms to EMFs. Volunteers in the sensitive group were selected on the basis of a self-report of being sensitive to EMFs, particularly those produced by mobile communication handsets and base stations. Volunteers in the control group confirmed that they were not sensitive to EMFs. Volunteers were excluded if they fulfilled one or more of the following criteria: were outside the age range of 18–80 years, had a history of brain injury, had a diagnosis of epilepsy or claustrophobia, had received a diagnosis or any treatment for a mental disease, had been fitted with a pacemaker, or had any physical impairment or illness or were taking any medication that could have, in some way, affected the results of the study. Particpants were recruited through local and national newspaper advertisements, letters of invitation, specialist health publications, personal recommendation, and Web site contact; they were reimbursed their travel expenses and received a small payment. All testing took place at a specialized Electromagnetics and Health Laboratory at the University of Essex (Colchester, UK) and was approved by the University of Essex Ethics Committee, the National Research Ethics Service, and the East of England Ambulance Service internal ethics group. All participants gave written informed consent before testing, which took place between April 2007 and January 2009.

### Design

A mixed design was used in which two groups (between subjects: sensitive and controls) were exposed to two exposure conditions (within subjects: TETRA and sham). Each participant took part in an open provocation session and two double-blind sessions. Each session was spaced at least 1 week apart and was conducted at about the same time of day. Subjective well-being and physiological functioning were measured during each session throughout the exposure conditions.

The initial open provocation session consisted of two tests where both the experimenter and the participant knew when the base station was “on” and when it was “off.” This was followed by a brief double-blind test comprising four short-duration trials with two on and two off conditions. Sessions 2 and 3 comprised the double-blind component of the study, with just one exposure (TETRA or sham) being administered during each of these sessions. All participants received both exposure conditions counterbalanced across sessions 2 and 3. Exposure conditions were block-randomized within group and preprogrammed into the exposure system control computer by an external consultant (Belloul 2008). Double-blinding remained in place until completion of the data collection phase.

Our sample size was calculated on the assumption that RF-EMFs has a small effect on human health (*d* = 0.40). Hence, 66 participants per group would yield statistical power of 0.90 for within-subjects effects, which allowed us to be 90% confident about differences found between sham and TETRA exposure conditions. The same level of confidence for between-subjects effects (i.e., group by exposure condition interaction) would require 132 participants per group.

### Materials and equipment: screened semianechoic chamber

**Figure 1:**
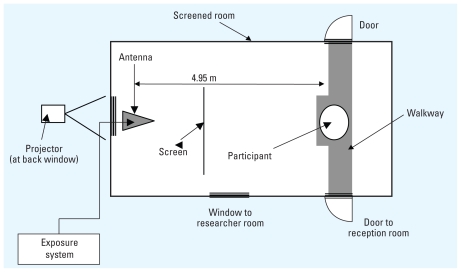
Diagram of the exposure chamber.

The testing room had a shielding effectiveness between 55 and 60 dB at 420 MHz. The appropriate depth absorber (300 mm) was used to achieve a uniform field at 420 MHz and conformed to the BS EN 61000-4-3:2006 “on” + A1: 2008 specification (National Physical Laboratory, Teddington, Middlesex, UK). Participants were seated 4.95 m from the antenna of the base station. A screen was placed 2.8 m from the participant, blocking the antenna from view, and was used to back-project task instructions for the participant during testing. The projector was positioned outside the testing room at the back wall, and projection was made through a screened window behind the antenna. A screened window in the near wall allowed visual contact between experimenter and participant (Figure 1).

#### Exposure system

We used the TETRA signal release 1 [specification 390 392–2; European Telecommunications Standards Institute (ETSI), Sophia-Antipolis Cedex, France], which comprised a time-division multiple-access frame structure with four time slots per frame on a single carrier. The chosen frequency was 420 MHz with a 25-kHz bandwidth. The signal was emitted at a power flux density of 10 mW/m^2^ (uncertainty estimate, 1 dB) over the area in which the participant was seated. Calculating a value for the Specific Absorption Rate (SAR) for whole body exposure is extremely complex; the variation in both the electrical properties of individual tissues and their spatial distributions means that SAR will never be uniform across the tissues of the body. In addition, SAR calculations should ideally be based on measurements using either a physical or numerical phantom, neither of which was available to us. Nevertheless, we would like to provide an estimated SAR for the present study. We calculated an approximate corresponding SAR for the power level used with this formula: body surface area × power flux density ÷ body weight. We used assumed values for body surface area and body weight; thus, for a person with a body surface area of 1.9 m^2^ and a body weight of 70 kg, the approximate corresponding SAR for this power level is 271 μW/kg (1.9 × 10mW/70 kg). The maximum power produced by TETRA base station transmitters is comparable to those of mobile phone base station transmitters (NRPB 2001). In the absence of any other available evidence, we broadly based the power level for this study on one conducted by Mann et al. (2000) who measured signal strengths of mobile phone base stations around the United Kingdom. With reference to the International Commission on Non-Ionizing Radiation Protection (1998) guidelines the power level used in the present study is 0.5% of the 2.1W/m^2^ reference level for the general public. The properties of the TETRA signal were modeled directly on the TETRA system that is currently used by the emergency services, thus replicating as closely as possible actual base-station emissions. Whether or not traffic is carried on the channel has a profound effect on the waveform. Thus, to allow for the harmonics of the time slots, a ratio of time slot occupancy of 50% traffic/no traffic was applied over the period of the tests. The sham condition comprised a no-signal condition. [A copy of the technical reference manual is available upon request (Belloul 2008).]

Regular calibrations confirmed that power density levels remained within 1 dB variability tolerance throughout the study period. The field uniformity was independently tested and verified by the National Physical Laboratory.

#### Biographical information

Participation began with a medical history interview, which provided biographical information and assessed participants’ state of health prior to testing.

#### Subjective well-being

Visual analog scales (VASs) and symptom scales were used to measure subjective well-being. The VAS each comprised a 10-cm line, with 0 cm representing “not at all” and 10 cm “extremely,” for anxiety, tension, arousal, relaxation, discomfort, and fatigue. The corresponding descriptors were “anxious,” “tense,” “agitated,” “relaxed,” “discomfort,” and “tired.” Participants were asked to mark anywhere on the line corresponding to how they felt at that time. The symptom scales were derived from the EHQ (Eltiti et al. 2007b) and comprised 57 symptoms on a 5-point scale (“not at all” to “a great deal”); participants were required to select how much they were suffering from each symptom.

#### Physiological measures

Physiological effects were measured using the means ± SDs for blood volume pulse (BVP), heart rate (HR), and skin conductance (SC) recorded throughout the open provocation and double- blind tests. We recorded physiological data using a ProComp Infiniti eight-channel encoder with Biograph Infiniti software [version 2.0.1; Thought Technology Ltd., Plattsburgh, New York, USA (2003)] that we ran on a Dell Latitude notebook (Dell Products UK, Dublin, Ireland). Signals were sampled at a rate of 2,048 samples/sec for BVP and 256 samples/sec for SC. The BVP was submitted to a fourth-order Butterworth low-pass filter with a 10-Hz cutoff frequency. The HR was calculated from the filtered BVP by calculating the time locations for the BVP peaks and valleys based on the locations on which the derivative of the BVP reached zero (dicrotic notches were ignored by the algorithm). HR was then estimated based on the time between peaks: HR = 1/(interpeak interval). All signals were resampled at 8 samples/sec to have a uniform rate. BVP signals were detrended because the important information in this signal was on the peak-to-peak values (Eltiti et al. 2007a).

#### “On”/“Off” judgments

There were six double-blind on/off judgments: four 5-min trials after the open provocation component and two 50-min trials. Participants were required to judge whether the base station was “on” or “off” and to indicate whether their confidence was “low,” “moderate,” or “high” directly after each trial.

#### Procedure

The open provocation test and the 5-min double-blind tests were conducted in session 1. Sessions 2 and 3 were double-blind and contained one exposure condition each. Ordering of exposure condition was randomized across participants. Table 1 shows the procedures in detail.

### Statistical analyses

We adjusted the alpha levels using the Bonferroni correction to control for type 1 error. Analysis of variance (ANOVA) was used to measure mean differences of data with normal distributions. Where the distributions were skewed and could not be normalized, we used nonparametric methods instead; Mann–Whitney *U*-tests were used for between-group comparisons, and Wilcoxon signed rank tests for within-group comparisons. Nonparametric tests lack the flexibility of ANOVAs, which calculate interaction effects. To mimic this type of analysis using nonparametric methods, we performed between-subjects analyses on difference scores.

## Results

### Biographical information

**Figure 2:**
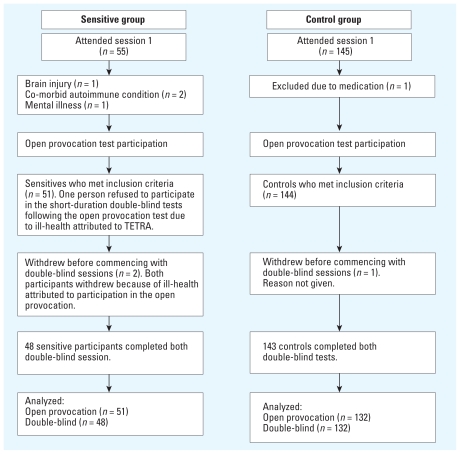
Flow of participants through each stage of testing. Five data sets from the 144 controls that completed the open provocation test were incomplete because of technical problems, and six males were randomly replaced with 6 females to achieve matching by sex. These 11 data sets plus the data set of the person who withdrew after session 1 (total of 12) were not analyzed.

Fifty-one “sensitive” participants and 144 control volunteers completed at least one session (Figure 2). However, during the testing, we lost data for five controls because of technical failures, and one person withdrew after session 1. Additionally, we tested controls more quickly than sensitives and found that the ratio of males to females was significantly higher for the controls compared with the sensitive group. Therefore, we tested an additional six females and randomly replaced six males (Figure 2). Our final sample of 183 (51 sensitives, 132 controls) included 10 emergency service workers, of these, two were sensitive participants. An independent-samples *t*-test showed that the sensitive groups (mean ± SD = 42 ± 16; range, 18–73) and control groups (41 ± 19; range, 18–80) were comparable in terms of age [*t*(181) = 0.289, *p* > 0.77] and sex [sensitives, 61% female; controls, 51% female; χ^2^ (1) = 1.487, *p* > 0.2].

The EHQ responses indicated that 11 (21.6%) sensitive participants reported being “a little bit” sensitive, 10 (19.6%) “moderately,” 11 (21.6%) “quite a bit,” and 12 (23.5%) “a great deal.” Six (11.8%) reported that they were not sensitive to EMFs but specified EMF-emitting objects as being associated with their symptoms. We conducted telephone interviews with these participants to establish eligibility to participate as sensitive before attending session 1. One person in the sensitive sample did not complete the EHQ before participating but was invited after a telephone interview. Assuming that any differences between these subgroups would be greatest under open provocation conditions, we performed Kruskal–Wallis tests on the open provocation physiological measures, VASs, and symptom scales. The outcomes for all these comparisons suggest that these groups were broadly comparable [(χ^2^ (4) ≤ 5.659, *p* > 0.28].

Some participants had comorbid chronic conditions. The most commonly reported condition in both groups was high blood pressure (sensitives, *n* = 2; controls, *n* = 4); asthma was also reported (sensitives, *n* = 1; controls, *n* = 1). One person in the sensitive group reported being “hypoglycemic,” and another reported suffering from both myalgic encephalomyelitis and irritable bowel syndrome. In the control group, chronic conditions reported were underactive thyroid (*n* = 3), type 2 diabetes (*n* = 2), type 1 diabetes (*n* = 1), arthritis (*n* = 1), bronchitis (*n* = 1), prostate problems (*n* = 1), back injury (*n* = 1), hernia (*n* = 1), and osteoporosis (*n* = 1). In addition, one participant reported being prone to gastroenteritis. Table 2 presents health comparisons between sensitive and control participants. Chi-square tests indicated a significant difference between the groups only for “headache proneness” [(χ^2^ (1) = 12.736, *p* < 0.001], with a greater percentage of sensitives reporting “headache proneness” than controls.

### Visual analog scales

We analyzed all VAS data using nonparametric statistics. We took baseline VAS measures before each test. However, this meant that for the open provocation test there was one baseline measure for both exposure conditions. We therefore included baseline scores only for the double-blind analyses, where one baseline measure served for one exposure condition.

In the open provocation test, we found an overall difference for group (sensitive vs. control) for all six variables (*p* ≤ 0.008), indicating that sensitives experienced greater anxiety, tension, arousal, discomfort, and fatigue and less relaxation than controls, regardless of exposure condition. The overall difference for exposure (TETRA, sham) was also significant for all variables (*p* < 0.001) except for fatigue (*p* = 0.037, α = 0.008), indicating that participants, independent of group, felt worse during TETRA compared with sham. Table 3 shows that, under TETRA conditions compared with sham, controls reported significantly higher levels of anxiety only. However, sensitive participants reported higher levels of anxiety, tension, arousal, and discomfort and lower levels of relaxation during TETRA compared with sham. Analyses of difference scores (TETRA – sham) revealed that sensitive participants reported a significantly greater increase in levels of anxiety, tension, arousal, and discomfort during the TETRA exposure compared with controls.

Under double-blind conditions, we found no significant overall difference between groups (*p* ≥ 0.1). For exposure condition, relaxation alone emerged as significant (*z* = −2.713, *p* = 0.007), with higher relaxation being reported during TETRA (mean ± SE, 6.76 ± 0.12) compared with sham (6.54 ± 0.11). Table 4 shows that, when controlling for baseline, however, both within- and between-group comparisons revealed no significant differences for any of the variables.

### Symptom scales

We also analyzed total symptom score, which measures symptom severity, and the total number of symptoms reported by means of nonparametric statistics.

During the open provocation test, we found an overall difference between groups for both the total symptom score (*z* = −5.985, *p* < 0.001) and total number of symptoms reported (*z* = −6.448, *p* < 0.001), indicating that sensitive participants experienced a greater severity (mean ± SE = 5.92 ± 1.23, *n* = 51) and number of symptoms (4.63 ± 0.86, *n* = 51) than did controls (1.04 ± 0.17 and 0.92 ± 0.13, respectively; *n* = 132). We also found an overall difference for exposure condition (TETRA, sham) for symptom severity only (*z* = −2.255, *p* = 0.024), suggesting that all participants experienced greater symptom severity when exposed to TETRA (2.89 ± 0.552) compared with sham (1.91 ± 0.322). The total number of symptoms reported remained comparable across exposure conditions (*z* = −1.874, *p* > 0.06). Control participants reported no differences between exposure conditions for either symptom severity or total number of symptoms reported (Table 5). In contrast, the sensitive participants reported a significantly higher total symptom score and more symptoms during TETRA than during sham. The between-group analyses of difference scores (TETRA – sham) revealed a significant difference for both total symptom score and total number of symptoms reported (Table 5).

Under double-blind conditions, we found a significant difference between groups for the total symptom score (*z* = −4.917, *p* < 0.001) and total number of symptoms reported (*z* = −5.282, *p* < 0.001), with sensitives reporting greater symptom severity (mean ± SE = 5.23 ± 1.06, *n* = 48) and number of symptoms (4.03 ± 0.71, *n* = 48) compared with controls (1.20 ± 0.18 and 0.92 ± 0.10, respectively; *n* = 132). We found no overall effect for exposure. Thus, sensitives reported a greater severity and number of symptoms compared with controls, regardless of exposure condition. Symptom reporting did not change as a function of exposure condition for either group, and between-group analyses revealed no significant differences (Table 5).

### Physiological measures

For the open provocation test, the mean HR data were normally distributed. The five remaining means and standard deviations data were positively skewed, thus requiring transformation. The distributions for the BVP mean and SD and HR SD data showed no improvement after transformation (Kolmogorov–Smirnov test, *p* ≤ 0.03), so we did not analyze them. We log_10_-transformed the remaining data. For the double-blind data, the BVP (mean) could not be transformed, and we did not analyze these data. HR (mean) data were normally distributed. We logarithmically transformed the remaining data (Table 6). We analyzed the data using a 2 × 2 exposure (sham, TETRA) × group (sensitive, control) ANOVA for open provocation and double-blind tests.

The ANOVA for HR (mean) produced a significant main effect for group (*p* = 0.008), indicating that sensitives had higher overall HR readings (mean ± SE = 71.72 ± 1.42) than did controls (67.26 ± 0.88) during the open provocation test. None of the ANOVA results for any other variables measured during the open provocation test were significant. Under double-blind conditions, ANOVA revealed a reliable between-group difference for HR (mean) only (*p* = 0.019). In general, HR readings were higher for sensitives (77.04 ± 1.43) than for controls (73.10 ± 0.861). However, after adjusting for type 1 error (α = 0.008), this finding was not significant. We found no other significant comparisons either within or between groups.

### “On”/“Off” judgments

**Figure 3:**
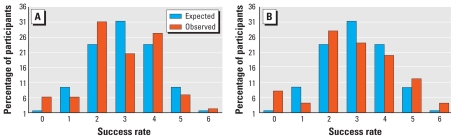
The probability of getting 0–6 of 6 correct: shown as a percentage by group of expected outcome versus observed outcome. (*A*) Controls (*n* = 132). (*B*) Sensitives (*n* = 48).

On four occasions judgment was required after 5 min of exposure (session 1) and on two other occasions after 50 min (sessions 2 and 3). We performed Pearson chi-square tests of successful judgments by trial × group. After correcting for multiple comparisons (α = 0.008), we found no significant differences (Table 7). Results that appeared significant at α = 0.05 did not show a consistent pattern across trials. Thus, neither group could tell above chance (50%) when the base station was “on” or when it was “off.” Figure 3 presents the binomial distribution with the expected rate of success (0 of 6 correct, 1 of 6 correct, etc.) at chance compared with the observed rate of success in each group. Two sensitives and three controls correctly judged all six trials. The distribution shows a slight response bias toward “off,” with controls tending to judge the base station as “off” 57% of the time and sensitive participants 51% of the time.

## Discussion

This investigation is the first to examine the short-term effects of a TETRA base station signal on human health and well-being. We found no evidence to suggest that TETRA base station signals have a negative impact on health and well-being in either the control or sensitive groups. In addition, neither group could reliably detect the presence of a TETRA signal. These findings concur with two recent studies conducted on TETRA handset signals (Barker et al. 2007; Green et al. 2005) as well as a growing body of evidence on GSM and UMTS signals, which found no differences between active and sham conditions and no ability to detect the presence of EMFs (Eltiti et al. 2007a; Kwon et al. 2008; Lyskov et al. 2001b; Regel et al. 2006; Rubin et al. 2006; Zwamborn et al. 2003). Moreover, Rubin et al. (2010) concluded in their systematic review that the evidence linking IEI-EMF to EMFs is weaker now than in their first review (Rubin et al. 2005). Taken together, these findings suggest that RF-EMFs in the range of approximately 400–2100 MHz do not have a negative impact on human health.

This study has some limitations. Our study tested individuals who identified themselves as sensitive to EMFs. Unlike a handset, which one can know to be on or off, reporting a sensitivity specifically to the TETRA base station signal is difficult, because it is unlikely that one can know where the signal is coming from. Therefore, this study addresses the general question of whether people with a perceived sensitivity to EMFs are affected by TETRA. We did not ascertain the exact onset of symptoms for each sensitive participant, and our study does not address long-term consequences of EMF exposure. We were not able to recruit the target number of sensitive participants in the 2.5-year period of funding for the study. Even with these caveats, we had sufficient power to detect a reliable difference in the open provocation condition. When participants knew they were being exposed to a TETRA signal, we found changes in most measures, but these results were not replicated under double-blind conditions. Sensitive participants continued to report more symptoms and experienced a greater overall severity of symptoms than did controls, but this effect was independent of exposure condition. This pattern of results replicates our previous findings using GSM and UMTS signals, which showed that only during open provocation conditions did sensitive participants report increased symptom and reduced well-being under active signal conditions (Eltiti et al. 2007a). Overall, these results suggest that it is not acute exposure to a base station signal that causes symptoms, but the knowledge of that exposure.

The long-term health consequences of EMF exposure are most often associated with physical health risks such as cancer. However, the long-term consequences of prolonged stress and anxiety that accompany the expectations associated with the everyday experiences of IEI-EMF individuals may be equally serious and, to our knowledge, have not been studied. For example, evidence suggests that ongoing worry is associated with low HR variability and increased HR during waking and sleeping, which, in turn, is associated with an increased risk of cardiovascular disease (Brosschot et al. 2007). Such findings indicate the importance of cognitive factors in IEI-EMF. Therefore, we recommend that future research should include a case–control study to elucidate the long-term impact of IEI-EMF on health.

## Figures and Tables

**Table 1:** Procedures for open provocation and double-blind tests.

	Task	Duration
Session 1		
Background information	Medical history interview, psychologic questionnaires, WAIS-R verbal memory test	1 hr
Open provocation (e.g., sham, TETRA)	VAS completed every 5 min, symptoms reported, physiologic measurements taken continuously	15 min for each exposure; 2-min “washout” between exposures
Cognitive tests	Participants completed Backward Digit Span Task and Letter Cancellation Task	8 min
Double-blind trials (e.g., sham, sham, TETRA, TETRA)	At the end of each of the four short-duration trials, participants specified whether they believed the base station had been “on” or “off” and how confident of this judgment they were	5 min for each exposure; 2-min “washout” between exposures

Session 2 and 3, double-blind (e.g., session 2, TETRA; session 3, sham)		
Exposure per session (TETRA/sham)	Physiologic measurements were taken continuously over the entire exposure period	50 min
Low load	Participants watched *Planet Earth* DVD, completed VAS every 5 min, and recorded any symptoms	20 min
High load	Participants performed an operation span task; the task was interrupted every 5 min to allow for the completion of VAS and symptom scales	20 min
Cognitive tests	Participants completed Backward Digit Span Task and Letter Cancellation Task	8 min
On/off judgment	Participants made a judgment as to whether the base station was “on” or “off”; procedure was identical to session 1	

WAIS-R, Wechsler Adult Intelligence Scale–Revised (Wechsler 1981). Standard cognitive tests were administered to assess whether the TETRA signal affects memory and attention. The results will be reported in a separate article.

**Table 2:** Additional health and social behavior information [*n* (%)].

	Yes		No		
Variable	Sensitive (*n*= 51)	Control (*n*= 132)	Sensitive (*n*= 51)	Control (*n*= 132)	χ^2^^a^
Chronic illness	5 (9.8)	15 (11.4)	46 (90.2)	117 (88.6)	0.002
Headache-prone^b^	24 (47.1)	25 (18.9)	27 (52.9)	100 (75.8)	11.9*
Taking medication	21 (41.2)	59 (44.7)	30 (58.8)	73 (55.3)	0.07
Electric shock^c^	15 (29.4)	23 (17.4)	34 (66.7)	109 (82.6)	2.99
Smoker	6 (11.8)	14 (10.6)	45 (88.2)	118 (89.4)	0
Consumes alcohol	41 (80.4)	108 (81.8)	10 (19.6)	24 (18.2)	0

a Values reflect χ^2^ with Yates continuity corrections; *p*-values with or without this correction are very similar.

b Missing data for 7 (5.3%) control participants.

c Missing data for 2 (3.9%) sensitive participants.

* *p* < 0.008 (Bonferroni corrected α = 0.008).

**Table 3:** Descriptives (means ± SEs) and statistical tests (*z*-scores) for the open provocation VAS for sensitive and control participants.

VAS measure	Sham		TETRA		Difference score^a^		TETRA vs. sham		Sensitive vs. control difference score^c^
	Sensitive	Control	Sensitive	Control	Sensitive	Control	Sensitive^b^	Control^b^	
Anxiety	1.51 ± 0.22	0.99 ± 0.10	2.24 ± 0.27	1.12 ± 0.11	0.72 ± 0.16	0.13 ± 0.07	−4.022#	−2.650#	−2.935#
Tension	1.51 ± 0.23	1.00 ± 0.10	2.31 ± 0.29	1.20 ± 0.12	0.80 ± 0.18	0.20 ± 0.06	−4.442#	−2.325*	−3.879#
Arousal	1.54 ± 0.23	0.96 ± 0.10	2.20 ± 0.27	1.12 ± 0.11	0.66 ± 0.17	0.16 ± 0.06	−4.170#	−1.527	−3.852#
Discomfort	1.92 ± 0.29	1.25 ± 0.11	2.71 ± 0.31	1.41 ± 0.13	0.79 ± 0.24	0.16 ± 0.06	−3.244#	−2.533**	−2.903#
Fatigue	3.05 ± 0.34	2.04 ± 0.17	3.18 ± 0.34	2.12 ± 0.17	0.13 ± 0.21	0.08 ± 0.10	−1.244	−1.628	−0.792
Relaxation^d^	7.44 ± 0.28	8.16 ± 0.15	6.65 ± 0.29	7.94 ± 0.16	−0.79 ± 0.24	−0.22 ± 0.08	−3.478#	−2.032*	−2.367*

All data were analyzed using corresponding *t*-tests with very similar results.

a Difference score mean ± SE: TETRA – sham; positive value indicates TETRA > sham.

b *z*, Wilcoxon signed rank test.

c *z*, Mann-Whitney *U*-test.

d Relaxation is reversed; therefore, a high score tends toward “extremely” relaxed.

* *p* ≤ 0.05.

** *p* ≤ 0.01.

# *p* < 0.008 (Bonferroni corrected α = 0.008).

**Table 4:** Descriptives (means ± SEs) and statistical tests (*z*-scores) for the double-blind VAS for sensitive and control participants.

VAS measure	Sham				TETRA				Difference score^a^		TETRA vs. sham		Sensitive vs. control difference score^c^
	Sham BL		Sensitive	Control	TETRA BL		Sensitive	Control					
	Sensitive	Control			Sensitive	Control			Sensitive	Control	Sensitive^b^	Control^b^	
Anxiety	1.46 ± 0.24	0.97 ± 0.09	1.95 ± 0.24	1.13 ± 0.08	1.18 ± 0.21	0.94 ± 0.09	1.63 ± 0.23	1.14 ± 0.09	−0.03 ± 0.37	0.04 ± 0.14	−0.144	−0.308	−0.128
Tension	1.52 ± 0.25	0.96 ± 0.09	2.06 ± 0.26	1.20 ± 0.09	1.23 ± 0.21	0.97 ± 0.10	1.73 ± 0.23	1.16 ± 0.09	−0.03 ± 0.36	−0.05 ± 0.05	−0.056	−0.110	−0.061
Arousal	1.41 ± 0.24	1.01 ± 0.10	2.12 ± 0.26	1.15 ± 0.09	1.16 ± 0.21	0.85 ± 0.09	1.77 ± 0.22	1.21 ± 0.11	−0.11 ± 0.37	0.22 ± 0.22	−0.056	−1.380	−0.636
Discomfort	1.42 ± 0.26	0.97 ± 0.09	2.07 ± 0.26	1.08 ± 0.09	1.09 ± 0.18	0.88 ± 0.09	1.67 ± 0.22	1.08 ± 0.10	−0.07 ± 0.32	0.09 ± 0.09	−0.210	−0.580	−0.639
Fatigue	2.17 ± 0.29	1.49 ± .014	2.70 ± 0.29	1.60 ± 0.13	2.25 ± 0.27	1.33 ± 0.13	2.58 ± 0.29	1.51 ± 0.13	−0.20 ± 0.45	0.07 ± 0.07	−0.338	−0.551	−0.615
Relaxation^d^	7.70 ± 0.26	8.39 ± 0.12	6.00 ± 0.25	6.74 ± 0.12	7.78 ± 0.26	8.40 ± 0.12	6.34 ± 0.24	6.91 ± 0.14	0.25 ± 0.48	0.16 ± 0.16	−0.072	−1.100	−0.670

BL, baseline. All data were analyzed using corresponding *t*-tests with very similar results.

a Difference score mean ± SE for double-blind tests = (TETRA – TETRA baseline) – (sham – sham baseline); positive value indicates TETRA > sham.

b Double-blind within-group analyses were performed on the difference between exposure and baseline scores using Wilcoxon sign rank tests [i.e., (TETRA – baseline) vs. (sham – baseline)].

c Double-blind between-group analyses were performed on the difference of the difference scores using Mann–Whitney *U*-tests; that is, sensitive (TETRA – baseline) – (sham – baseline) vs. control (TETRA – baseline) – (sham – baseline).

d Relaxation is reversed; therefore, a high score tends toward “extremely” relaxed.

**Table 5:** Symptom scales descriptives (means ± SEs) and statistical tests (*z*-scores) for the open provocation and double-blind tests for sensitive and control participants.

Test	Sham		TETRA		Difference score^a^		TETRA vs. sham		Sensitive vs. control difference score^c^
	Sensitive	Control	Sensitive	Control	Sensitive	Control	Sensitive^b^	Control^b^	
Open provocation									
Total symptom score	3.94 ± 1.00	1.13 ± 0.19	7.90 ± 1.75	0.95 ± 0.19	3.96 ± 1.44	−0.17 ± 0.18	−3.586*	−0.973	−4.957*
Total number of symptoms reported	3.55 ± 0.80	1.03 ± 0.16	5.71 ± 1.06	0.80 ± 0.13	2.16 ± 0.74	−0.23 ± 0.14	−3.783*	−1.852	−5.798*
Double-blind									
Total symptom score	6.63 ± 1.65	1.25 ± 0.22	3.84 ± 0.88	1.15 ± 0.19	−2.78 ± 1.56	−0.11 ± 0.21	−1.266	−0.637	−1.291
Total number of symptoms reported	5.06 ± 1.04	0.91 ± 0.13	2.99 ± 0.67	0.92 ± 0.12	−2.07 ± 1.03	0.01 ± 0.15	−1.578	−0.640	−1.363

a Difference score: TETRA – sham; positive value indicates TETRA > sham.

b Wilcoxon signed rank tests.

c Mann-Whitney *U*-tests.

* *p* ≤ 0.001.

**Table 6:** Descriptives (means ± SEs for original untransformed data) and statistical tests (*F*-scores) for physiologic measures for sensitive and control participants by exposure during open provocation and double-blind tests.

Test/measure	Sham		TETRA		Condition	Sensitive vs. control	Group × condition
	Sensitive	Control	Sensitive	Control			
Open provocation							
BVP mean^a^	36.02 ± 0.03	36.10 ± 0.06	36.01 ± 0.03	36.13 ± 0.10	NA	NA	NA
BVP SD^a^	1.15 ± 0.13	1.12 ± 0.09	1.10 ± 0.13	1.14 ± 0.11	NA	NA	NA
SC mean	6.99 ± 0.58	7.61 ± 0.44	6.99 ± 0.55	7.66 ± 0.45	0.24	0.06	0.45
SC SD	0.93 ± 0.11	0.95 ± 0.07	0.94 ± 0.12	0.99 ± 0.08	0.08	0.12	0.08
HR mean	71.78 ± 1.50	67.58 ± 0.88	71.66 ± 1.46	66.94 ± 0.88	2.11	7.16**	1.02
HR SD^a^	7.46 ± 0.73	7.11 ± 0.59	7.00 ± 0.78	6.84 ± 0.54	NA	NA	NA
Double-blind							
BVP mean^a^	36.30 ± 0.14	36.71 ± 0.49	36.23 ± 0.08	36.16 ± 0.06	NA	NA	NA
BVP SD	1.19 ± 0.09	1.58 ± 0.35	1.17 ± 0.12	1.27 ± 0.08	0.18	0.15	0.20
SC mean	9.50 ± 0.88	8.87 ± 0.47	8.84 ± 0.74	7.98 ± 0.40	1.03	0.92	0.42
SC SD	1.63 ± 0.13	1.68 ± 0.09	1.62 ± 0.11	1.49 ± 0.09	0.78	0.45	3.34
HR mean	77.01 ± 1.32	73.51 ± 1.08	77.07 ± 1.29	72.69 ± 0.91	0.25	5.59*	0.33
HR SD	9.40 ± 0.79	8.83 ± 0.59	10.63 ± 1.16	8.23 ± 0.47	0.04	3.17	1.11

NA, not applicable. Nonparametric statistics were also performed on the untransformed data with virtually the same results (copies of this analysis are available upon request).

a These data did not lend themselves to transformation because participants’ scores were tightly grouped around the mean; therefore, ANOVAs were not conducted on these data.

* *p* ≤ 0.05.

** *p* ≤ 0.01.

**Table 7:** Successful and failed judgments by trial for sensitive and control participants [*n* (%)].

Trial	Sensitive (*n*= 50)			Control (*n*= 132)			Sensitive vs. control χ^2^^a^
	Correct	Incorrect	χ^2^^a^	Correct	Incorrect	χ^2^^a^	
Trial 1^b^	26 (52.0)	24 (48.0)	0	66 (50.0)	66 (50.0)	0	0.01
Trial 2^b^	28 (56.0)	22 (44.0)	0.33	61 (46.2)	71 (53.8)	0.54	1.03
Trial 3^b^	31 (62.0)	19 (38.0)	2.17	55 (41.7)	77 (58.3)	3.82*	5.23*
Trial 4^b^	25 (50.0)	25 (50.0)	0	55 (41.7)	77 (58.3)	2.96	0.71
Trial 5^c^,^d^	22 (45.8)	26 (54.2)	0.08	72 (54.5)	60 (45.5)	0.97	0.75
Trial 6^c^,^d^	16 (33.3)	32 (66.7)	4.2*	69 (52.3)	63 (47.7)	0.1	4.34*

a Values reflect χ^2^ with Yates continuity corrections. *p*-Values with or without the correction are very similar.

b Duration of exposure, 5 min.

c Duration of exposure, 50 min.

d Sensitive group: *n* = 48 for sessions 2 and 3.

* *p* ≤ 0.05.
